# Best evidence topic: Can acute appendicitis manifest with normal inflammatory markers?

**DOI:** 10.1016/j.amsu.2020.09.003

**Published:** 2020-09-08

**Authors:** Rashid Ibrahim, Pushpa Veeralakshmanan, James Ackah, Pedram Panahi

**Affiliations:** Department of General Surgery University Hospitals Plymouth NHS Trust, Plymouth, PL6 8DH, United Kingdom

**Keywords:** Inflammatory markers (IM), Acute appendicitis (AA), C-reactive protein (CRP), White cell counts (WCC)

## Abstract

A best evidence topic has been constructed using a described protocol. The three-part question addressed was: for patients with suspected acute appendicitis can normal inflammatory markers rule out the diagnosis? Altogether 151 papers were found using the search strategy reported below. Seven were identified to provide the best evidence to answer the question. The author, journal, date, and country of publication, patient group studied, study type, relevant outcomes, results, and study weaknesses were tabulated. In conclusion, six out of seven papers are more in favour with the concept that normal inflammatory markers cannot effectively rule out the diagnosis of acute appendicitis.

## Highlights

• The aim is to asses the incidence of normal inflammatory markers among patients with acute appendicitis.
• Seven articles were included in this best evidence topic.
• Six articles agreed that normal inflammatory markers cannot exclude a diagnosis of acute appendicitis.

## 1 Introduction

This Best Evidence Topic (BET) was devised using a framework outlined by the International Journal of Surgery [1]. This format was used because a preliminary literature search showed that the available evidence has insufficient quality and is too homogenous to conduct a meaningful meta-analysis. A BET provides evidence based answers to common clinical questions, using a systematic approach to reviewing the literature.

## 2 Clinical scenario

A 25-year-old female presents with a history of shifting right iliac fossa pain for two days, she has normal inflammatory markers and a negative pregnancy test. An abdominal ultrasound was not conclusive for appendicitis. You question whether normal inflammatory markers can rule out appendicitis and safely discharge the patient.

## 3 Three part question

In [patients with suspicion of acute appendicitis], Can [normal inflammatory markers] rule out [a diagnosis of acute appendicitis]?

## 4 Search strategy

Medline 1946 to May 2020 and Embase 1974 to May 2020 using the OVID interface:

[Acute appendicitis] AND [normal inflammatory markers OR normal C-reactive protein OR normal CRP OR normal white cells counts OR normal WCC].

Medline using the PubMed interface:

[Acute appendicitis] AND [normal inflammatory markers OR normal C-reactive protein OR normal CRP OR normal white cells counts OR normal WCC].

The results were limited to English articles and human studies.

## 5 Search outcome

**Fig. 1:**
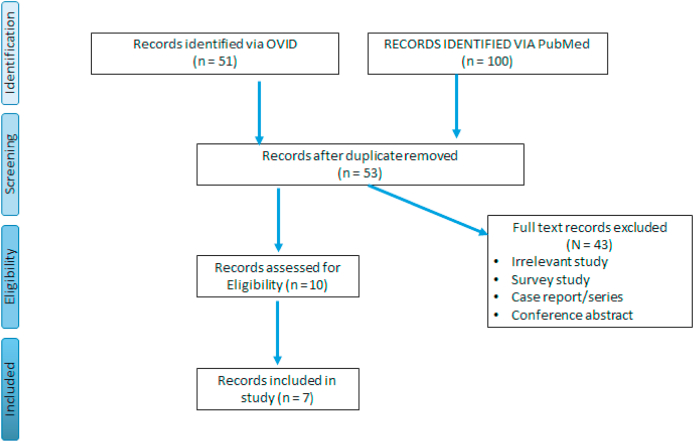
PRISMA flow chart.

A total of 151 papers were found using OVID and PubMed interface. A total of 53 papers were identified after we removed duplicates. Out of these, 43 papers were excluded based on titles and abstracts. Ten full-text articles were screened and assessed for eligibility. From these, seven papers were identified that provided the best evidence to answer the question. The search strategy process is detailed in Fig. 1. Eligible patients were defined as those presenting with both normal WCC and CRP with a histologically confirmed acute appendicitis. In addition to the false-negative result of these inflammatory markers, most of the included papers also investigated the sensitivity, specificity, positive and negative predictive values. However, we have mainly focused on the false-negative result because the rest is out of the scope of this best evidence topic.

## 6 Results

**Table:** 

Author, date of publication, journal and country, study type (level of evidence)	Patient group	Outcomes	Key results	Additional comments
Shefki Xharra et al. [2] 2012 World Journal of Emergency Surgery Kosovo a prospective double blinded clinical study level II	Study included 173 patients who operated on for suspected AA Between November 2008 to February 2009 Age range (5–59) years 148 (85.5%) patients had histology proven acute appendicitis	Percentage of patients with normal WCC + CRP among those who have histology proven AA	9 patients (6%) with confirm AA out of 148 with normal IM.	Single centre study No description of randomisation process No report about the duration of follow up Risk of selection bias by including only patients with suspected AA NO matching between duration of symptoms and level of inflammatory markers

**Table:** 

Jasper J. Atema et al. [3] (2015), Academic Emergency Medicine Sweden and Netherlands, Retrospective review of five cohort studies. (Level III)	Study included 1024 patients with clinically suspected AA Between February 1997 and June 2000 Age: only adult patients 18 year old and above 580 patients had final diagnosis of AA 101 out of 1024 had both normal WCC + CRP	Percentage of patients with normal WCC + CRP among those who have histology proven AA	12 patients (11.8%) out of 101 had normal WCC + CRP and proven AA on histology	Largest number of study population in the literature Study included only adult patients Retrospective study Risk of incorporation bias: (final diagnosis as assigned by expert panel) Risk of verification bias because surgical findings and histopathological Examination was not available for some patients. Selection bias: including only patients with suspected AA High prevalence of AA in the study limit the external validity of our results. NO matching between duration of symptoms and level of inflammatory markers
Jason J. Y. Kim. et al. [4] (2019), ANZ Journal of Surgery Australia Multicentre, prospective, observational study (Level III)	949 patients in 27 centres underwent appendectomy for suspected AA between June and October 2016 Age: adult + paediatrics 749 had histology proven AA patients with normal inflammatory markers in relation to symptoms: Group A – normal WCC/CRP <24 HR (n = 33) Group B – normal WCC/CRP 24-48HR (n = 51) Group C normal WCC/CRP > 48HR (n = 44)	Percentage of patients with normal WCC + CRP among those who have histology proven AA	A: 48.5% B: 35.3% C: 38.6% Over all 39.8% patients with normal WCC + CRP have histology proven AA	Highest false negative result reported in literature Risk of selection bias: only including patients who have undergone Appendicectomy Variable inflammatory marker concentration cut-off values used in previous studies, can make conclusions difficult Lack of blinding of recruiting doctors.
PG Vaughan-Shaw. et al. [5] (2016) Journal of the Royal Society of Medicine Short Reports, UK, Retrospective cohort study (Level III)	297 patients with acute appendicitis who underwent Appendicectomy were included from two centres Centre A 113 patients Centre B 184 patients Age range: adults and paediatrics November 2005 to October 2006 (centre A) April 2009 to May 2010 (centre B). WCC + CRP + NC (neutrophil count) were studied as parameter for IM	Percentage of patients with normal inflammatory markers who have histology proven appendicitis	Centre A: 4 patients 3.5% Centre B: 13 patients 7% Over all result in both centres 6.3%	Retrospective study Risk of selection Bias Included only patient with acute appendicitis NO matching between duration of symptoms and level of inflammatory markers
Nalin H. Dayawansa [6] 2016 Australia ANZJSurg Retrospective cohort study (Level III)	Total 400 patients who underwent Appendicectomy March 2012 and September 2014 Age: adult patients above 16 281 patients had histologically proven appendicitis,	Percentage of patients with normal inflammatory markers who have histology proven appendicitis	24 (8.54%) of patient with acute appendicitis had normal CRP and WCC on presentation	Retrospective study single centre incomplete documentation of clinical features include only adults No matching between duration of symptoms and level of IM Risk of selection Bias Included only patients with suspected AA
JM Grönroos [7] 2001 Finland Acta Pñ diatr Study type not mentioned ?case control study	200 children with suspicion of acute appendicitis divided into 2 groups Group A: 100 had uninflamed appendix at histology Group B: 100 had inflamed appendix at histology From 1995 to 1999 Age range 0–18 years Mean age 11 years	Percentage of patients with normal inflammatory markers who have histology proven appendicitis in children	In children within the AA group: normal WCC + CRP (7%)	Study type not mentioned Single centre Limited to paediatric age group No matching between duration of symptoms and level of IM
Anshuman Sengupta Et al [8] 2009 Ann R Coll Surg Engl UK Prospective study (Level III	98 patients with lower abdominal suspected of acute appendicitis were included December 2006 to February 2007 out of this 22 underwent Appendicectomy, 6 had diagnostic laparoscopy without removal of the appendix Remaining 70 patients were discharge or referred to other specialities	Percentage of patients with normal inflammatory markers who have histology proven appendicitis	No patients with both CRP + WCC within the normal range had acute appendicitis, giving a sensitivity and a negative predictive value of 100%	Nothing mention about methods of exclusion of appendicitis in 70 patients. Nothing mentioned about the duration of follow up for those 70 patients No correlation between duration of symptoms and level of inflammatory markers Study population number is very small. Single centre Age range or median was not mentioned

## 7 Discussion

Shefki Xharra et al. [2] conducted a prospective double-blinded clinical study in 2012 to assess the accuracy of IM in the diagnosis of acute appendicitis: the false-negative result of both CRP and WCC was 6%. The conclusion was that the combination of CRP and WCC has greater diagnostic accuracy in acute appendicitis. This significantly decreases false positive and false negative diagnoses, but none of these is 100% diagnostic for acute appendicitis. In 2015, Jasper J. Atema et al. [3] conducted a large multicentre retrospective review of five cohort studies of 1024 adult patients with clinically suspected AA who presented with a duration of symptoms ranging from 2 h to 5 days were included. They found 12 patients (11.8%) among those with normal IM had a final diagnosis of appendicitis. The conclusion was: no WCC count or CRP level can safely and sufficiently confirm or exclude the suspected diagnosis of acute appendicitis in patients who present with abdominal pain of 5 days or less in duration. Jason J. Y. Kim et al. [4] recently published a multicentre prospective observational study in 2019 to evaluate the relationship between normal IM, duration of symptoms, and proven appendicitis. Of interest was the finding that a total of 38.9 of patients with normal CRP and WCC had appendicitis. Therefore, the study concluded that normal inflammatory markers can't exclude appendicitis, even in those with prolonged duration of symptoms. PG Vaughan-Shaw et al. [5] in 2016 also reached the same conclusion after they conducted a retrospective two independent cohort studies to assess the relationship between normal IM and AA, they added neutrophils count in addition to WCC and CRP, they reported AA with normal inflammatory markers in 6.3% of the patients. They disagree with the view of Sengupta et al. [8]. Who suggests that patients with normal WCC and CRP are unlikely to have AA. Nalin H. Dayawansa et al. [6] reported an 8.54% rate of acute appendicitis with normal IM in their Case-control retrospective analysis which included 400 adult patients. Also, in 2001 JM Grönroos [7] conducted what was seems to be a case-control study which included 200 paediatric patients with suspected acute appendicitis and reported a 7% incidence of normal WCC and CRP among patients with AA.

In contrast to all of the above-mentioned studies which showed increasing evidence that normal IM markers are not effectively helpful to exclude AA, there is one study conducted by Anshuman Sengupta et al. [8] in 2009, which retrospectively reviewed 98 patients presenting with lower abdominal pain 21 patients had Appendicectomy with acute appendicitis confirmed in 19 patients. Six patients had diagnostic laparoscopy with a normal appendix (not removed). The remaining 70 patients were either discharged home or referred to other specialties. in the study nothing was mentioned about the method of exclusion of acute appendicitis among those 70 patients. The results showed no patients with both WCC and CRP within the normal range had acute appendicitis, giving sensitivity and a negative predictive value of 100%. So the conclusion was: patients experiencing lower abdominal pain, with normal WCC and CRP values, are unlikely to have acute appendicitis and can be safely sent home.

## 8 Clinical bottom-line

Out of these seven studies, six have supported the concept that normal inflammatory marker levels cannot “rule out” acute appendicitis. Furthermore, the authors recommend relying on a combination of clinical signs, images and serial measurements of inflammatory markers to exclude a diagnosis of acute appendicitis.

## 9 Provenance and peer review

Not commissioned, externally peer reviewed.

## Author contribution

Pushpa Veeralakshmanan: assisted in the data collection, analysis and writing of the paper. James Ackah and Pedram Panahi: assisted in writing of the paper.

## Declaration of competing interest

There are no conflicts of interest.
